# Characterization of a vacuolar sucrose transporter, HbSUT5, from *Hevea brasiliensis*: involvement in latex production through regulation of intracellular sucrose transport in the bark and laticifers

**DOI:** 10.1186/s12870-019-2209-9

**Published:** 2019-12-27

**Authors:** Xiangyu Long, Heping Li, Jianghua Yang, Lusheng Xin, Yongjun Fang, Bin He, Debao Huang, Chaorong Tang

**Affiliations:** 1 Key Laboratory of Biology and Genetic Resources of Rubber Tree, Ministry of Agriculture, Haikou, Hainan People’s Republic of China; 2 College of Tropical Crops, Hainan University, Haikou, 570228 Hainan China; 3 Subtropical Agriculture Research Institute, Fujian Academy of Agricultural Sciences, Zhangzhou, 363005 Fujian China

**Keywords:** Sucrose transporter, Laticifer, Vacuole, Latex production, Rubber tree

## Abstract

**Background:**

Sucrose (Suc), as the precursor molecule for rubber biosynthesis in *Hevea brasiliensis*, is transported via phloem-mediated long-distance transport from leaves to laticifers in trunk bark, where latex (cytoplasm of laticifers) is tapped for rubber. In our previous report, six Suc transporter (SUT) genes have been cloned in Hevea tree, among which *HbSUT3* is verified to play an active role in Suc loading to the laticifers. In this study, another latex-abundant SUT isoform, *HbSUT5*, with expressions only inferior to *HbSUT3* was characterized especially for its roles in latex production.

**Results:**

Both phylogenetic analysis and subcellular localization identify HbSUT5 as a tonoplast-localized SUT protein under the SUT4-clade (=type III). Suc uptake assay in baker’s yeast reveals HbSUT5 to be a typical Suc-H^+^ symporter, but its high affinity for Suc (Km = 2.03 mM at pH 5.5) and the similar efficiency in transporting both Suc and maltose making it a peculiar SUT under the SUT4-clade. At the transcript level, HbSUT5 is abundantly and preferentially expressed in Hevea barks. The transcripts of *HbSUT5* are conspicuously decreased both in Hevea latex and bark by two yield-stimulating treatments of tapping and ethephon, the patterns of which are contrary to HbSUT3. Under the ethephon treatment, the Suc level in latex cytosol decreases significantly, but that in latex lutoids (polydispersed vacuoles) changes little, suggesting a role of the decreased *HbSUT5* expression in Suc compartmentalization in the lutoids and thus enhancing the Suc sink strength in laticifers.

**Conclusions:**

Our findings provide insights into the roles of a vacuolar sucrose transporter, HbSUT5, in Suc exchange between lutoids and cytosol in rubber-producing laticifers.

## Background

Natural rubber (*cis*-polyisoprene) is a kind of crucial industrial raw material, and its commercial source is exclusively from a tropical tree species, *Hevea brasiliensis* (para rubber tree). As in all plants, photosynthetic carbohydrates produced in the leaves are essential energy sources for growth and metabolism of Hevea trees. Sucrose (Suc) is the major transport form of photosynthetic carbohydrates from the leaves to peripheral tissues [1], and also serves as a substrate for rubber biosynthesis in the cytoplasm (latex) of specialized phloem rubber-producing laticifers [2, 3]. Translocation of Suc to various sinks is mediated by Suc transporters (SUTs) [4–7], which are membrane proteins with twelve transmembrane spanning domains [8, 9]. SUTs are normally divided into five clades (SUT1 to 5) or three types (I to III; type I = SUT1 clade, type II = SUT2, SUT3 and SUT5 clades, type III = SUT4 clade), by specific physiological characteristics and functions [6, 7, 10–13].

In regularly exploited rubber trees, the laticifers in trunk bark represent a major Suc sink, and Suc concentration in latex is positively correlated with rubber yield under some circumstances [14–18]. The vital role of a SUT1-clade member, *HbSUT3* (synonym: *HbSUT1B*) in Suc loading to laticifers and consequentially in rubber productivity, have been previously demonstrated [19, 20]. To increase our understanding of the regulatory networks of Suc loading, transport and storage in rubber-producing laticifers, it is necessary to characterize the other *HbSUTs* expressed in these cells. In the latex of Hevea trees, the expression of a SUT4-clade member, *HbSUT5,* was only lower than *HbSUT3* among the six *SUT* genes [19, 21]. Most plant species seem to have only one SUT4 member, and their physiological roles have attracted much attention in recent years. Interestingly, most of the SUT4 clade members characterized to date have been assigned to the vacuolar membrane (tonoplast), regulating networks of Suc loading, transport and storage in vacuoles [22–26]. In the laticifers of Hevea trees, lutoids, as a polydispersed system of vacuoles, comprise nearly 20% of the latex volume, and are critical in controlling the duration of latex flow after tapping, thereby influencing latex yield [27]. Lutoids are also thought to function in storage and detoxification, and have lysosomal and osmotic roles. Therefore, characterizing the physiological roles of the potential lutoid membrane-localized SUT member HbSUT5 is of great interest. In the present study, the functions of HbSUT5 were investigated by subcellular localization, *in planta* expressions under different conditions, and Suc uptake kinetics in baker’s yeast, in order to define a possible physiological role in latex production.

## Results

### Subcellular identification of *HbSUT5*

**Fig. 1:**
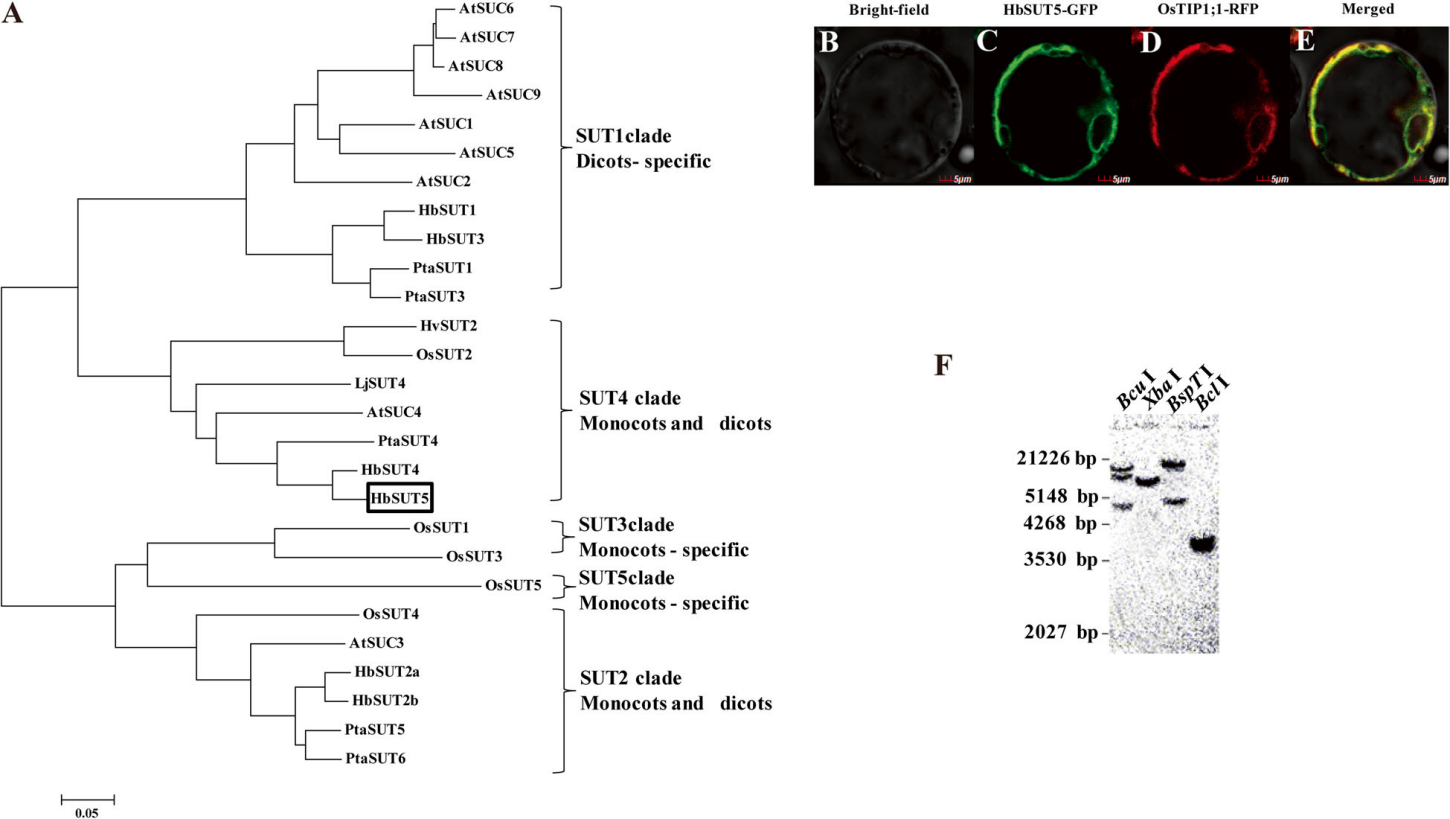
HbSUT5 as a single or low copy vacuolar *SUT* gene. **a**. Phylogenetic tree of HbSUT5 and some selected plant sucrose transporters. Full length amino acid sequences of SUTs were exploited to create the phylogenetic tree by MEGA4. The GenBank accession numbers for the peptide sequences of other SUTs are At1g71880 (AtSUC1), At1g22710 (AtSUC2), At2g02860 (AtSUC3), At1g09960 (AtSUC4), At1g71890 (AtSUC5), At5g43610 (AtSUC6), At1g66570 (AtSUC7), At2g14670 (AtSUC8), At5g06170 (AtSUC9), BAA24071 (OsSUT1), BAC67163 (OsSUT2), BAB68368 (OsSUT3), BAC67164 (OsSUT4), BAC67165 (OsSUT5), ADW94615 (PtaSUT1), ADW94616 (PtaSUT3), ADW94617 (PtaSUT4), ADW94618 (PtaSUT5), ADW94619 (PtaSUT6), CAD61275 (LjSUT4), CAB75881 (HvSUT2), ABJ51933 (HbSUT1), ABJ51934 (HbSUT2A), ABJ51932 (HbSUT2B), ABK60190 (HbSUT3), ABK60191(HbSUT4) and ABK60189 (HbSUT5). **b-e**. Localization of HbSUT5-GFP and OsTIP1;1-RFP in rice protoplasts (Scale bar = 5 μm). **b**. Bright field image; **c**. Transient expression of GFP; **d**. Transient expression of RFP; **e**. Merged GFP and RFP image. **f**. DNA gel blot analysis of the HbSUT5 under high stringency. Genomic DNA from Hevea was digested with restriction analysis, resolved on a 0.8% agarose gel (20 μg/lane), and blotted to Hybond N^+^, and hybridized with a ^32^P-labled 528-bp-long *HbSUT5* cDNA fragment

The *H. brasiliensis SUT* (*HbSUT*) gene, *HbSUT5*, was cloned previously [19, 21], but has not been further characterized. *HbSUT5* contained a 1497-bp-long ORF that predicted a protein of 498 amino acids with theoretical molecular weight of 54.1 kDa and pI of 9.39. Phylogenetic analysis (Fig. 1a) revealed that HbSUT5, together with another Hevea tree SUT, HbSUT4, belongs to SUT4 clade SUTs. As predicted by the method of TMHMM (http://www.cbs.dtu.dk/services/TMHMM/) [28], HbSUT5 consists of 12 transmembrane spans, short cytoplasmic N- and C-terminals, and a short central cytoplasmic loop between transmembrane spans 6 and 7, the characters of which are typical of the SUT4 clade SUT members (Additional file 2: Figure S1) [5]. To further verify the subcellular locations of HbSUT5, the HbSUT5 protein fused with GFP (green fluorescent protein) was transiently expressed in rice protoplasts together the known rice tonoplast intrinsic protein OsTIP1;1 fused with RFP (red fluorescent protein) [29]. The GFP signals overlapped completely with the RFP signals (Figs. 1b-e), indicating that HbSUT5 shares a similar subcellular location with the well-characterized tonoplast protein OsTIP1;1. High-stringency DNA gel blot analysis showed that there are one to three hybridization bands under different restriction analyses, indicating *HbSUT5* to be a single or low copy gene in the Hevea genome (Fig. 1f), the results of which has been further verified by our high-quality draft genome of the rubber tree [30].

### Functional analysis of *HbSUT5* in baker’s yeast

**Fig. 2:**
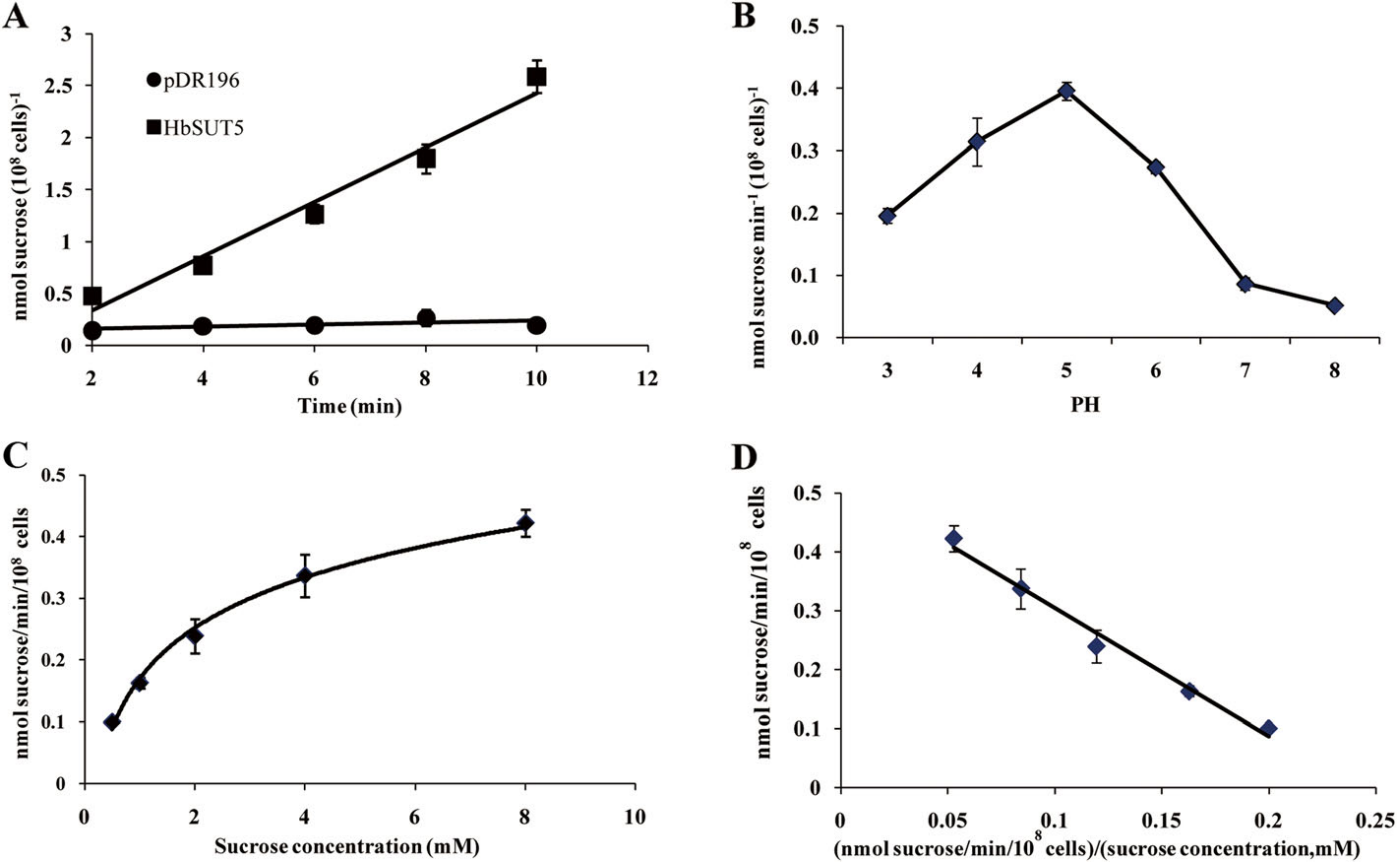
Characterization of the sucrose transport function of HbSUT5 in baker’s yeast *Saccharomyces cerevisiae* SEY6210. **a**. Time course of sucrose uptake; **b**. Effects of pH on sucrose uptake; **c**. Concentration-dependent uptake; **d**. An Eadie-Hofstee plot representing the mean rate of uptake as a function of sucrose concentration

To test whether HbSUT5 has Suc transport activity*,* assays with baker’s yeast cells expressing the *HbSUT5* cDNA were performed using radiolabeled ^14^C-Suc. Suc transport into yeast cells expressing the *HbSUT5* was found to be nearly linear within the assayed time course (2 to 10 min), reaching approximately 13-fold higher than that of the control yeast harboring the empty vector pDR196 after 10 min (Fig. 2a). These results demonstrated that *HbSUT5* encodes a functional Suc transporter.

Suc transport kinetics analysis displayed that the transport activity of HbSUT5 was obviously dependent on the pH of the buffer solution, showing the highest at pH around 5.0 but decreasing progressively with the elevation of buffer pH (Fig. 2b). The Suc affinities of HbSUT5 were measured at pH 5.5 under a Suc substrate range of 0.5–8 mM, and the data were used to perform the Michaelis-Menten analysis (Fig. 2c). The Eadie-Hofstee double-reciprocal plots of the data (Fig. 2d) revealed that the estimated Km was 2.03 mM with a transport rate Vmax of 0.522 Suc min^− 1^ (10^8^ cells)^− 1^, suggesting HbSUT5 belongs to a high-affinity/low-capacity (HALC) Suc transporter [31].

The substrate specificity of HbSUT5 was detected with several unlabeled sugars as ^14^C-Suc competitors, including galactose, fructose, inositol, lactose, Suc, maltose and raffinose. The results showed that only Suc and maltose could significantly compete at similar extents with the ^14^C-Suc uptake (Table 1), indicating the relative substrate specificity of HbSUT5 in transporting sugars. In addition, three sugars, i.e., fructose, inositol and lactose revealed to stimulate Suc uptake somewhat. Further, Suc transport by HbSUT5 was strongly inhibited by the protonophores of carbonyl cyanide *m*-chlorophenylhydrazone (CCCP) and dinitrophenol (DNP) (Table 1). This result together with the character of pH-dependent Suc transport ability (Fig. 2b) clearly indicated that HbSUT5 was a H^+^-Suc symporter.

**Table 1:** Effect of competing sugars and inhibitors on sucrose uptake by HbSUT5 expressed in SEY6210 yeast

Treatment	% Activity*
Competing sugars	
Control	100
10 mM Galactose	101 ± 7.5
10 mM Frucose	121 ± 9.5^a^
10 mM Inositol	130 ± 15.9^a^
10 mM Lactose	132 ± 6.2^a^
10 mM Sucrose	42 ± 2.6^A^
10 mM Maltose	44 ± 3.4^A^
10 mM Raffinose	110 ± 7.0
Inhibitors	
Control	100
50 μM CCCP	86 ± 5.5 ^a^
50 μM NEM	89 ± 6.9 ^a^

*Analysis of variance (ANOVA) by SAS 6.12, where *P* < 0.01 was marked by A, and *P* < 0.05 by a

### *In planta* expression patterns of *HbSUT5*

**Fig. 3:**
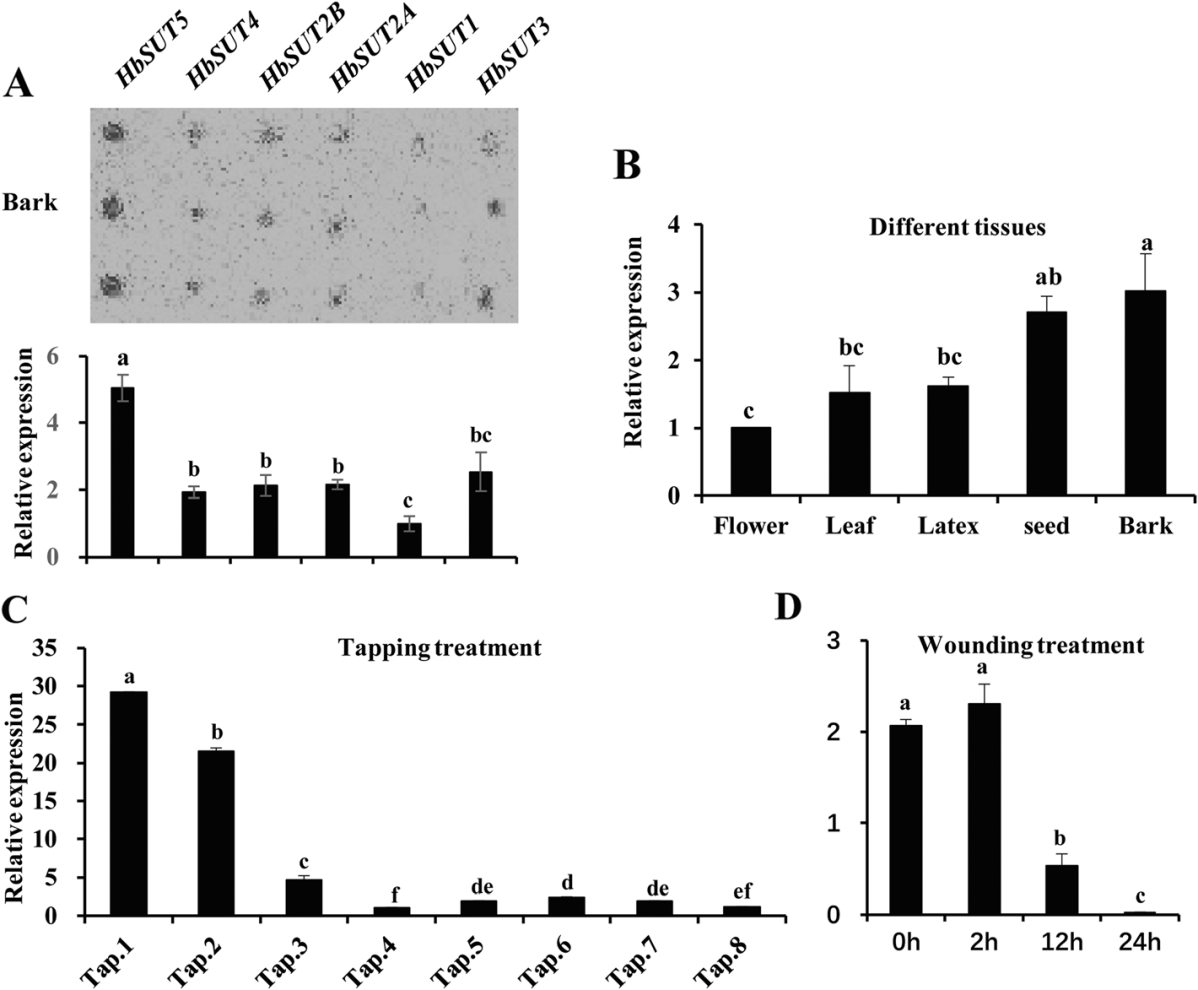
Expressional characterization of HbSUT5. **a, b**. Expression of *HbSUTs* in different *Hevea* tissues. **a**. Expression comparison of different *HbSUTs* in bark was measured by reverse Northern blot analysis; The relative transcript levels were obtained by scanning the intensity of reverse Northern blots. **b**. Tissue-specific expression patterns of *HbSUT5* were measured by QPCR. **c**, **d**. Expression of *HbSUT5* in response to tapping and wounding treatments by QPCR. Effect of tapping (1st to 8th) (**c**) and wounding (0 to 24 h) (**d**) treatments on *HbSUT5* expression was conducted in mature virgin rubber trees. (Statistical analysis: significant difference, *P* < 0.01, marked by different lowercase)

As reported previously, among the six *HbSUT* genes, *HbSUT5* is abundantly expressed in the latex, only inferior to *HbSUT3*, and shows much higher expression than that of the other SUT4-clade SUT gene, *HbSUT4* [19]. Besides being an abundant *SUT* isoform in the latex, *HbSUT5* was the predominant isoform in the bark (Fig. 3a), predicting the multifunctional roles of this gene. The transcript abundance of *HbSUT5* was then compared by QPCR (quantitative polymerase chain reaction) in five Hevea tissues, including leaf, latex, seed, flower and bark. As shown in Fig. 3b, *HbSUT5* was most highly expressed in the bark and seed, followed by the latex and leaf, and the lowest in the flower.

**Fig. 4:**
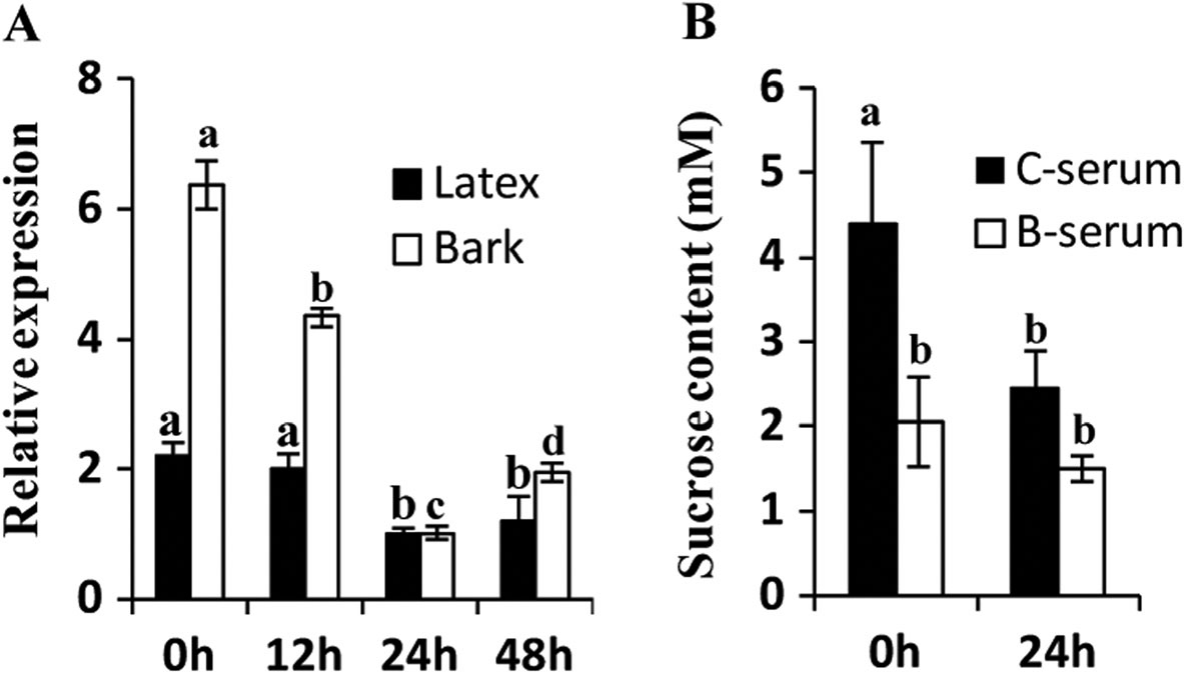
HbSUT5 influences the sucrose content. **a**. The expression of HbSUT5 was examined in the latex and bark tissues after 1% ethephon bark treatment of regularly tapped rubber trees. **b**. The sucrose content was measured between cytosol (C-serum) and lutoid (B-serum) at 24 h after 1% ethephon treatment. (Statistical analysis: significant difference, P < 0.01, marked by different lowercase)

Increased Suc loading to the laticifers is one of the most important mechanisms responsible for ethylene-stimulated latex production [19, 32, 33]. To further investigate the functions of *HbSUT5*, its expression patterns were analyzed in the latex and bark tissues by QPCR after ethephon (2-chloroethylphosponic acid, an ethylene releaser) bark treatment. *HbSUT5* expression was significantly down-regulated both in the latex and bark tissues, attaining at 24 h post treatment about 40% of its initial level in the latex and 15% in the bark (Fig. 4a). To further probe the roles of decreased HbSUT5 expression under the ethephon treatment, the Suc contents were examined in latex cytosol (C-serum) and lutoids (B-serum). The results showed that the C-serum Suc was decreased significantly by the treatment, but the B-serum Suc was much less affected, suggesting a function of the lowered HbSUT5 expression in reducing Suc export from lutoids (Fig. 4b). The tapping of virgin (never tapped before) Hevea trees is an ideal model to identify and characterize latex regeneration-related genes due to the conspicuous stimulating effect of tapping on latex production for the first few tappings [19, 34]. As shown in Fig. 3c, the expressions of *HbSUT5* in the latex markedly depressed with the first four consecutive tappings, reaching less than 4% of its initial level, and maintaining low levels thereafter, the pattern of which correlates negatively with the conspicuous increase in latex yield with the tappings [19]. Bark wounding that stimulates latex production [35] was also found to decrease *HbSUT5* expression (Fig. 3d), reaching the lowest level at 24 h after treatment. The decreased *HbSUT5* expression in the latex by tapping was hypothesized to function in a similar mechanism as it did under the ethephon treatment.

### Characterization of the *HbSUT5* promoter

**Fig. 5:**
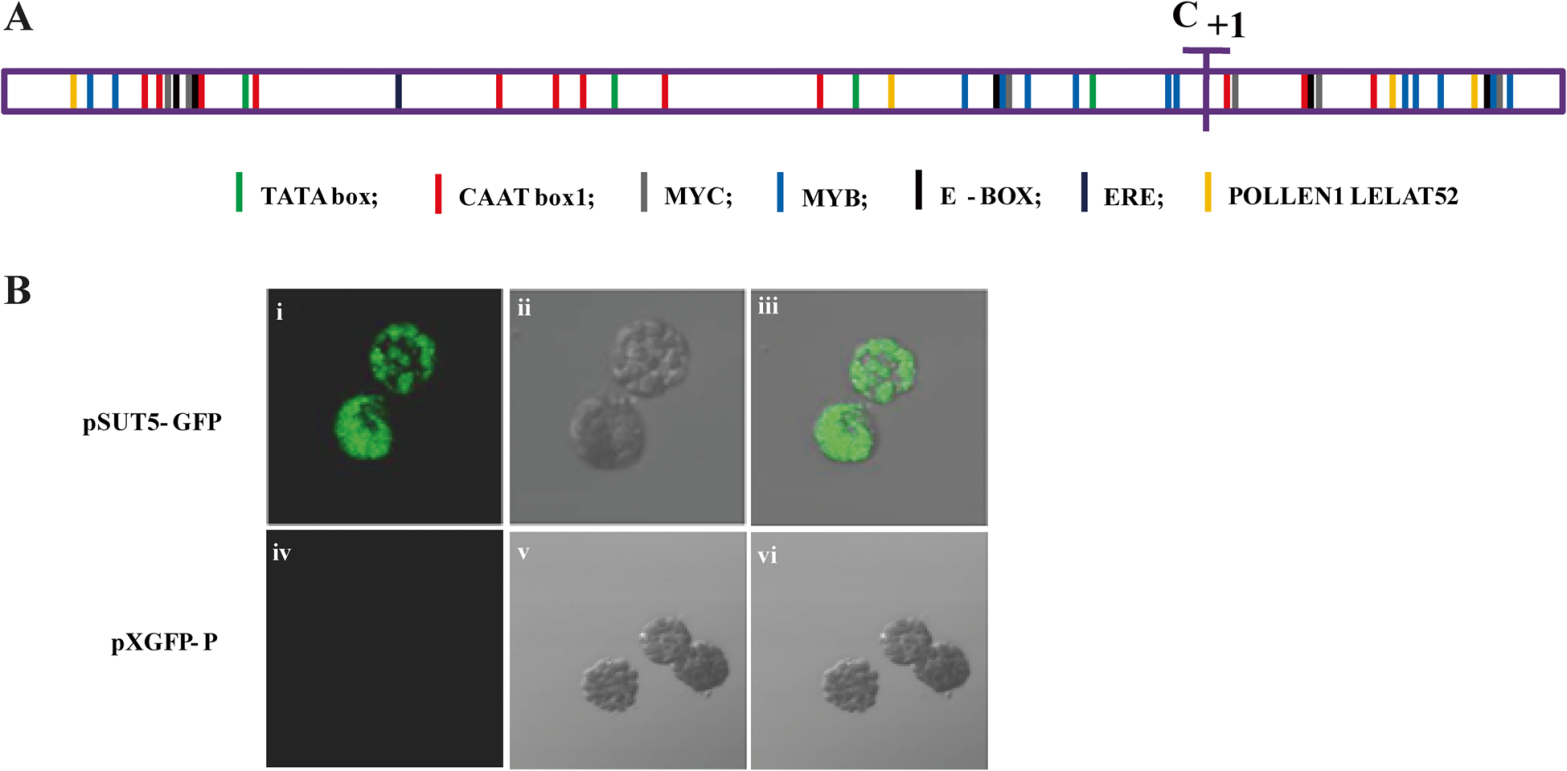
Characterization of the HbSUT5 promoter. **a**. Diagram of the promoter sequences of the *HbSUT5*. The promoter of HbSUT5 was analyzed for putative *cis*-acting sequences using the PLACE software (http://www.dna.affrc.go.jp/PLACE/). The putative transcriptional start site was highlighted by “C_+ 1_”. **b**. Transient expression analysis of the putative *HbSUT5* promoter in tobacco leaf protoplast. Fluorescent microscopy of GFP expression driven by the putative *HbSUT5* promoter (pSUT5-GFP) (i to iii) compared with the control GFP expression (pXGFP-P) (iv to vi) in tobacco leaf protoplast

To explore the transcriptional characters of *HbSUT5*, its putative promoter sequence of about 1.5-kb-long genomic sequence upstream from its start codon (GenBank: KU529197) [36] was characterized by in-silico analysis and heterogeneous transient expression. As revealed by the NNPP (neural network promoter prediction) promoter predictor (http://www.fruitfly.org/seq_tools/promoter.html), this sequence contained a high score (0.99) transcription promoter with a putative transcriptional start site (marked as C_+ 1_) located 342 bp upstream of the start codon (ATG) (Fig. 5a). The PLACE (plant cis-acting regulatory DNA elements) software (http://www.dna.affrc.go.jp/PLACE/) was then used to analyze the putative *cis*-acting elements harbored by the promoter. A total of 54 distinct cis-regulatory elements were predicted, many of which are implicated in multiple biological processes, including stress and hormone responses, signaling pathways, and tissue-, organ- or metabolism-specific expressions (Fig. 5a; Additional file 1: Table S1). The harboring of multiple minimal elements such as four TATA box and 21 CAAT box motifs suggested its capability to initiate accurate basal transcription in most plant species. Experimentally, its promoter activity was confirmed by the transient promoter expression analysis, which revealed a strong GFP fluorescence in tobacco leaf protoplasts transfected with the GFP expression cassette driven by the putative *HbSUT5* promoter (pSUT5-GFP) (Fig. 5b).

It is noteworthy that three classical ethylene-responsive elements (EREs) were predicted in the *HbSUT5* promoter, with one (− 785) on its sense orientation, and the other two (− 767 and − 327) on its antisense orientation. Besides that, there were 9 cis-acting elements with more than ten locations in the *HbSUT5* promoter, among which were those involved in dehydration response (MYB (myeloblastosis) and MYC (myelocytomatosis) recognition sites) [37, 38], tissue- or organ-specific expression (E-Box “CANNTG”, POLLEN1 element “AGAAA” and tetranucleotide “YACT”) [39, 40, 41], overall expression activation (CT-rich motif “TCTCTCTCT”, GATA-Box and ARR1 recognition site “NGATT”) [42–44], and sugar metabolism (DOF core recognition site “AAAG”) [45].

## Discussion

In higher plants, transportation, distribution and accumulation of photosynthetic carbohydrate are important and essential biochemical processes for growth and development. The photosynthetic carbon assimilates, mainly in the form of Suc, are distributed through phloem loading, long-distance translocation and unloading to diverse sinks [46]. Suc transporters (SUTs) have been demonstrated to be actively involved in these processes, and thus play an important physiological role in carbohydrate partitioning to regulate all known stages of plant growth and development [47–49].

In this study, a *H. brasiliensis* SUT4-clade (=type III) SUT member, *HbSUT5*, was functionally characterized by subcellular localization study, Suc-uptake assay (Fig. 2 and Table 1) and a number of *in planta* expressional analyses under a couple of latex-stimulating treatments (Figs. 3 and 4c, d). The *HbSUT5* has 12 transmembrane spanning domains (Additional file 2: Figure S1), showing typical structural characters of the GPH (Glycoside-Pentoside-Hexuronide) cation symporter family within the major facilitator superfamily [9]. Phylogenetic (Fig. 1a) and subcellular localization studies (Figs. 1b-e) further identify *HbSUT5* as a tonoplast-localized SUT4-clade SUTs. The SUT4-clade SUTs usually have lower affinity for Suc and belong to low-affinity/high-capacity transporters [23, 50–53]. As revealed by the Suc transport assay (Fig. 2), *HbSUT5* is a typical Suc-H^+^ symporter as most other SUT4-clade SUTs [23]. However, HbSUT5 has a high affinity for Suc (*K*m = 2.03 mM at pH 5.5), the character of which differs from most of the SUT4-clade SUT members in higher plants that catalyze Suc uptake with low affinity [7]. The only other SUT4-clade SUT reported to have high Suc-affinity is from carrot, DcSUT1 [54]. In contrast to the ubiquitous tissue expression of *HbSUT5* (Fig. 3a, b), the expression of *DcSUT1* is restricted in the green part of carrot plants, functioning in phloem Suc loading [54]. In addition to Suc and maltose, the SUT4-clade SUTs can transport a greater variety of glucosides than the SUT2-clade SUTs, but are more specific than the SUT1-clade SUTs [6]. For example, a SUT4-clade SUT from *Lotus japonicus*, LjSUT4 has broad substrate specificity for glucosides including Suc, salicin, helicon, maltose, sucralose and both α- and β-linked synthetic phenyl gucosides [23]. Of the seven mono-, di- or tri-saccharides examined, HbSUT5 efficiently transported only Suc and maltose (Table 1). It remains to be determined whether the substrates of HbSUT5 also include some of the glucosides transported by LjSUT4 and other SUT4-clade SUTs [6]. It is also note worthy that HbSUT5 transports maltose as efficiently as it does for Suc (Table 1), and all other characterized SUTs have been found to transport Suc more efficiently than maltose [6].

In mature plant cells, the central vacuole occupies 80 to 90% of the cell volume and as a sink for accumulation of ions, sugars and a plethora of other metabolites to regulate cellular pressure, detoxification and ecological interactions [55–57]. In Hevea laticifers, vacuoles appear in a polydispersed lysosomal vacuome, i.e. lutoids with storage functions that account for 10 to 20% by volume of fresh latex (essentially laticiferous cytoplasm) [58]. In recent years, the tonoplast-localized SUTs have been shown to play a regulative role in transporting Suc from the vacuole lumen to the cytoplasm, and function in multiple aspects of plant growth and development [23, 24, 59]. In *Lotus japonicus*, LjSUT4 is mainly expressed in roots and nodules, and its expression correlates positively with the process of nodulation [26, 59, 60]. In *Arabidopsis*, *AtSUT4* shows higher levels of expression in sink tissues, and functions in sucrose-induced inhibition of seed germination [61]. Using RNA interference (RNAi) in *Populus*, PtaSUT4 reveals to modulate both Suc export and its utilization in lateral and terminal sinks, and plays an important role in regulating whole-plant water relations, water stress response, and photosynthesis [25, 52]. Inhibition of *StSUT4* expression by RNAi leads to increased tuber yield and early flowering in transgenic potato plants, and its expression was also induced by gibberellin and ethephon [62].

In Hevea tree, latex regeneration in laticifers exploits Suc as the precursor molecule for rubber biosynthesis, which represents a high-carbon cost activity [58]. The efficiency of Suc uptake and catabolism in the laticifers is thus crucial for rubber productivity [14, 35]. It is now clear that these processes are controlled mainly by a Suc transporter, HbSUT3 [19], and a neutral/alkaline invertase, HbNIN2 [63], respectively. Tapping has a stimulating effect on latex production in virgin rubber trees, the latex yield of which increases conspicuously during the first 7 to 10 tappings [19]. The expressions of both *HbSUT3* [19] and *HbNIN2* [63] are markedly up-regulated by tapping in the latex, indicating an enhanced Suc uptake and catabolism that contributes to latex regeneration. In contrast, the expressions of the tonoplast *HbSUT5* in the latex decreased rapidly during the first four tappings, and then retained a low level thereafter (Fig. 3c). We hypothesize that the depression of *HbSUT5* expression contributes to tapping-stimulated latex production from two aspects. First, down-regulation of *HbSUT5* expression may result in decreased Suc exporting from lutoids into cytosol, and thus enhance further the (Suc) sink strength already stimulated by the activated Suc catabolism in the latex. Second, tapping enhanced Suc compartmentalization in the lutoids, which may benefit the integrity of lutoids and thus the duration of latex flow [27]. In regularly tapped Hevea trees, ethephon bark treatment increases latex production although to a lesser extent compared with the effect of tapping on virgin rubber trees [58]. *HbSUT5* expression was down-regulated by the ethephon treatment, although not as conspicuous as the tapping treatment (Fig. 3e), which may contribute to latex production in a similar mechanism. This hypothesis is evidenced by examining the Suc levels in latex and its cytosol and lutoids under the ethephon treatment. The marked decrease in the Suc levels both in latex [19] and its cytosol (Fig. 4b) highlights the insignificant change in lutoids Suc. The lowered *HbSUT5* expression may function in reducing Suc export from the lutoids. Interestingly, expression of *HbSUT5*, the trunk bark-dominant SUT isoform (Figs. 3a and b), was also significantly down-regulated by the ethephon treatment in the trunk bark. It remains to be determined in what ways such a regulation affects the Suc concentrations outside the laticifers, and thus the Suc uptake of laticifers. Analysis of *HbSUT5* promoter indicates the presence of multiple cis-acting elements implicated in response to various stresses (Fig. 5a). It is remarkable that the *HbSUT5* promoter harbors more than ten locations of dehydration-responsive cis-acting elements (Fig. 5a), possibly accounting for the conspicuous effect of tapping on *HbSUT5* expression. Tapping, the process of latex harvesting, results in a dehydration stress upon the rubber-producing laticifers, and the expressional responses of various functional genes contribute to harvesting-regulated latex regeneration [64]. Besides that, several ethylene and gibberellin response elements reside in the promoter, indicating the involvement of *HbSUT5* in ethylene and gibberellin pathways similar as a potato homolog, *StSUT4* [62].

## Conclusions

In this study, we have analyzed another Suc transporter gene, *HbSUT5*, in Hevea tree. HbSUT5 is a vacuolar Suc transporter that belong to SUT4-clade (=type III) and has high affinity for Suc. HbSUT5 is a bark-dominant SUT isoform, but also abundantly expressed in the latex (cytoplasm of laticifers). Contrary to the latex-dominant isoform, *HbSUT3*, *HbSUT5* expressions are obviously decreased by the tapping and ethephon treatments, indicating a negative response to two ways of yield stimulation. Combining the results of Suc content in latex and its cytosol and lutoids (polydisperse vacuoles), HbSUT5 is thought to function in Suc exchange between lutoids and cytosol in laticifers, and thus, together with HbSUT3 influences rubber yield formation in Hevea.

## Methods

### Plant materials

Reyan7–33-97 (synonym: CATAS7–33-97 or RY7–33-97) rubber trees (*H. brasiliensis*) were Hevea cultivars in Hainan Province, and provided by the experimental plantation of CATAS (Chinese Academy of Tropical Agricultural Sciences, Danzhou, Hainan, China). Selected trees were tapped under a S/2 d/3 system (a half spiral pattern, every three days). Five tissues, flower, leaf, latex, seed and bark, were collected for RNA extraction from Reyan7–33-97 with tapping for two years. The same type of trees was also used to treat with ethephon*.* The 8-year-old mature virgin (never tapped) trees were selected to treat with tapping and wounding.

### Ethylene, wounding and tapping treatment

For ethylene and wounding treatment, four batches of sixty trees were selected to detect the expression pattern of *HbSUT5,* and every batch had three biological duplications*.* In ethylene treatment, one batch was treated with 1% carboxyl methyl cellulose (CMC) as a control, and the other three batches were treated with 1% ethephon (2-chloroethylphosponic acid, an ethylene releaser) in 1% CMC at 12, 24 and 48 h before tapping. For the wounding treatment, three batches were wounded with pushpin at 2, 12 and 24 h before tapping, and the fourth batch was unwounded as the control. According to tapping treatment, fifteen virgin trees were divided into three biological duplications to tap for the first eight tapping. All treated trees were tapped at the same time, and the latex and bark were collected for RNA isolation. The details for ethylene, wounding and tapping treatments and sample collection were as described previously [19]. Besides that, the sucrose content was also measured in latex including cytosol (C-serum) and lutoid (B-serum) after ethephon treatment, using the anthrone method [19].

### RNA isolation, cDNA synthesis and QCR

Total RNA was extracted and treated with DNase I (TaKaRa, China), and examined to concentration and quality by NanoDrop 2000 (Thermo, USA) as described previously [65]. The PrimeScript™ II 1st Strand cDNA Synthesis Kit (TaKaRa, China) were used to cDNA systhesis following the manufacturer’s protocol. The primer pairs of *HbSUT5* (5′-AGTTGCTGGATAAGCTAGAGA-3′ and 5′-CGGTGGTCTAGCCCTG-3′) were used to analyze the expression patterns*,* and *YLS8* (mitosis protein) as the internal control gene for normalization [66]. The QPCR was operated by CFX96 Touch Real-Time PCR (Bio-Rad, USA), using the SYBR® Premix Ex Taq™ II (Perfect Real Time) (TaKaRa, China). The PCR reaction system, procedures and data analysis were to follow as the previous research [19].

### Southern and reverse northern blot

Genomic DNA of Reyan7–33-97 was extracted from mature leaves using the CTAB DNA extraction method. One pair of primers (the sense primer: 5′-AACAAAGAAACAGTTGCTGGATAAG-3′ and the reverse primer: 5′-CCTGAGTCATGTTATTAGCCAC-3′) was designed based on the cDNA and genomic sequences of *HbSUT5*, and used to amplify an intron-free fragment of 530-bp long that contains no enzyme cutting sites of *Bcu* I, *Xba* I, *BspT* I and *Bcl* I. The amplified fragment was digoxigenin-labeled with the Random Primer DNA Labeling Kit (TaKaRa, China), and used as the probe in subsequent Southern blot analysis. The 10 μg genomic DNA was digested by *Bcu* I, *Xba* I, *BspT* I and *Bcl* I overnight. The digested product was separated on 0.8% agarose gel and transferred onto a Hybond-N+ membrane (Amersham Pharmacia, USA). And the protocols of Southern blot analysis were performed according to the detailed instructions [67]. The Reverse Northern blot assays were performed to analyze the relative transcript abundance of the six *HbSUT* genes in bark as described previously [19].

### Subcellular localization

For subcellular localization analysis, the *HbSUT5* was sub-cloned with *Bsa* I site into the pBWA(v)HS-gfp vector to produce HbSUT5-GFP fusions. The gene encoding a known tonoplast intrinsic protein, *OsTIP1;1*, was sub-cloned with *Bsa* I site into pBWD (LB)-mKATE vector to produce OsTIP1;1-RFP fusions. The specific primer pairs used were 5′-CAGTGGTCTCACAACATGGCAATCCCACAGGCGGA-3′ and 5′-CAGTGGTCTCATACATGGGAGGGCCATGGGCTTCT-3′ (*HbSUT5*) and 5′-CAGTGGTCTCACAACATGCCGATCCGCAACATCGC-3′ and 5′-CAGTGGTCTCATACAGTAGTCGGTGGTGGGGAGCT-3 (*OsTIP1;1*). The protoplasts from leaves which rice seedlings had been cultured at 30 °C in darkness after germination for 7 to 15 days were used in transient expression analysis. The two genes with different label signals, HbSUT5 and OsTIP1;1, were co-transformed into protoplasts according to Yu et al. [68]. The transformed protoplasts were observed with laser scanning confocal microscope (Olympus FV1000, Janpan). The excitation and emission wavelengths were 480 and 510 nm for GFP, and 588 and 635 nm for RFP, respectively.

### Suc uptake assay in yeast

To understand characteristics of Suc transport, the amplified products of HbSUT5 were cut with *Sma* I and *Sal* I and cloned into the yeast expression vector pDR196, using the pair primers of 5′-GAGCCCGGGATGGCAATCCCACAG-3′ and 5′-GAACGTCGACTCATGGGAGGGCCA-3′ (underlined sequences for *Sma* I and *Sal* I recognition sites). The constructed vectors were then transformed into the baker’s yeast (*Saccharomyces cerevisiae*) strain SEY6210 for ^14^C-Suc uptake analysis according to the references [19, 69]. The statistical analysis of data was performed by SAS (statistical analysis system).

### Analysis of the *HbSUT5* promoter

The *HbSUT5* promoter, an approximate 1.5-kb fragment of the 5′-upstream region of the *HbSUT5* gene, had been previously isolated [36], and predicted for its cis-acting elements using the software of PLACE (www.dna.affrc.go.jp/htdocs/PLACE/). The *HbSUT5* promoter was amplified using gene-specific primers of 5′-CGACGGCCCGGGCTGGTA-3′ and 5′-TCCCTTCTCCTCGAAAGAAACAC-3′, and inserted into the transient expression vector of pXGFP-P that harbors the reporter gene GFP designed for transient promoter expression analysis [70]. The resulting construct, named as pSUT5-GFP, was transformed into tobacco protoplast using *Agrobacterium*-mediated approach, and the GFP fluorescence signal was scanned and detected after transformation for 24 h by a confocal laser scanning microscope (Olympus FV1000, Janpan). The details for the preparation of tobacco protoplast, *Agrobacterium*-mediated transformation and fluorescence signal scanning, were as described previously [71, 72].

## Supplementary information

**Additional file 1: Table S1.** Putative cis-regulatory elements of the HbSUT5 promoter predicted by using the PLACE software

**Additional file 2: Figure S1**. Prediction of the transmembrane spans for HbSUT5. The prediction was performed using the method of TMHMM (Krogh et al., 2001)

## Data Availability

All data generated or analyzed during this study are included in this published article and its supplementary information files. The authors are pleased to share the data upon request.

## Abbreviations

CCCP: Protonophores of Carbonyl Cyanide m-Chlorophenylhydrazone
CMC: Carboxyl Methyl Cellulose
DNP: Dinitrophenol
EREs: Ethylene Responsive Elements
GFP: Green Fluorescent Protein
GPH: Glycoside-Pentoside-Hexuronide
HALC: High-Affinity/Low-Capacity
HbNIN2: Neutral/alkaline invertase
MYB: Myeloblastosis
MYC: Myelocytomatosis
NNPP: Neural Network Promoter Prediction
ORF: Open Reading Frame
PLACE: Plant cis-acting regulatory DNA elements
QPCR: Quantitative Polymerase Chain Reaction
RFP: Red Fluorescent Protein
RNAi: RNA interference
Suc: Sucrose
SUT: Suc Transporter
TIP: Tonoplast Intrinsic Protein
