# Correction to: Characterization of a vacuolar sucrose transporter, HbSUT5, from *Hevea brasiliensis*: involvement in latex production through regulation of intracellular sucrose transport in the bark and laticifers

**DOI:** 10.1186/s12870-020-02748-y

**Published:** 2021-01-18

**Authors:** Xiangyu Long, Heping Li, Jianghua Yang, Lusheng Xin, Yongjun Fang, Bin He, Debao Huang, Chaorong Tang

**Affiliations:** 1 Key Laboratory of Biology and Genetic Resources of Rubber Tree, Ministry of Agriculture, Rubber Research Institute, Chinese Academy of Tropical Agricultural Sciences, Haikou, 571101 Hainan China; 2 College of Tropical Crops, Hainan University, Haikou, 570228 Hainan China; 3 Subtropical Agriculture Research Institute, Fujian Academy of Agricultural Sciences, Zhangzhou, 363005 Fujian China

**Correction to: BMC Plant Biol (2019) 19:591**

**https://doi.org/10.1186/s12870-019-2209-9**

In the original publication [1] there was an incomplete affiliation. In this correction article the correct and incorrect affiliation are published. The original article has been updated.

**Incorrect affiliation**

- Key Laboratory of Biology and Genetic Resources of Rubber Tree, Ministry of Agriculture, Haikou, Hainan, People’s Republic of China.

**Correct affiliation**

- Key Laboratory of Biology and Genetic Resources of Rubber Tree, Ministry of Agriculture, **Rubber Research Institute, Chinese Academy of Tropical Agricultural Sciences**, Haikou, **571101**, Hainan, China
