# Supplementary material for: Characterization of a vacuolar sucrose transporter, HbSUT5, from Hevea brasiliensis: involvement in latex production through regulation of intracellular sucrose transport in the bark and laticifers
Source: BMC Plant Biol. 2019 Dec 27;19:591. doi: 10.1186/s12870-019-2209-9 (PMC6935173; doi:10.1186/s12870-019-2209-9)
Supplement: Supplementary file 1 — Additional file 1: Table S1. Putative cis-regulatory elements of the HbSUT5 promoter predicted by using the PLACE software [file 12870_2019_2209_MOESM1_ESM.doc]

**Table S1 Cis-regulatory elements predicted on the HbSUT5 promoter**

| **Factor or Site Name** | **Location** | **Signal Sequence** | **Function** | **PubMed No.** |
| --- | --- | --- | --- | --- |
| -300ELEMENT | -646  [plus 5(-) strand sites] | TGHAAARK | an enhancer element for the endosperm-specific expression | [2152160](http://www.ncbi.nlm.nih.gov/entrez/query.fcgi?db=PubMed&cmd=Retrieve&list_uids=2152160&dopt=Citation) |
| 2SSEEDPROTBA | -115 | CAAACAC | Conserved in many storage-protein gene promoters; May be  important for high transcription activity | [8818291](http://www.ncbi.nlm.nih.gov/entrez/query.fcgi?db=PubMed&cmd=Retrieve&list_uids=8818291&dopt=Citation) |
| ACGTATERD1 | -53  [plus 1(-) strand sites] | ACGT | etiolation-induced expression of *erd1* (early responsive to dehydration) in *Arabidopsis* | [12535340](http://www.ncbi.nlm.nih.gov/entrez/query.fcgi?db=PubMed&cmd=Retrieve&list_uids=12535340&dopt=Citation) |
| ANAERO1CONSENSUS | -222, -14 | AAACAAA | One of 16 motifs found in silico in promoters of 13 anaerobic genes involved in the fermentative pathway (anaerobic set 1) | [16027132](http://www.ncbi.nlm.nih.gov/entrez/query.fcgi?db=PubMed&cmd=Retrieve&list_uids=16027132&dopt=Citation) |
| ANAERO2CONSENSUS | -244, -209 | AGCAGC | One of 16 motifs found in silico in promoters of 13 anaerobic genes involved in the fermentative pathway (anaerobic set 1) | [16027132](http://www.ncbi.nlm.nih.gov/entrez/query.fcgi?db=PubMed&cmd=Retrieve&list_uids=16027132&dopt=Citation) |
| ARR1AT | -244, -222, -209, -14  [plus 6(-) strand sites] | NGATT | Recognition site of *ARR1*, a response regulator; *Arabidopsis* *ARR1* and *ARR2* response regulators operate as transcriptional activators | [11135105](http://www.ncbi.nlm.nih.gov/entrez/query.fcgi?db=PubMed&cmd=Retrieve&list_uids=11135105&dopt=Citation) |
| ATHB1ATCONSENSUS | -939  [plus 1(-) strand sites] | CAATWATTG | Recognition sequence of *Arabidopsis* *Athb-1* protein with a HD-Zip motif | [8253077](http://www.ncbi.nlm.nih.gov/entrez/query.fcgi?db=PubMed&cmd=Retrieve&list_uids=8253077&dopt=Citation) |
| ATHB5ATCORE | -939 | CAATNATTG | Consensus binding sequence for *Arabidopsis* (A.T.) class I HD zip protein, ATHB5 | [16055682](http://www.ncbi.nlm.nih.gov/entrez/query.fcgi?db=PubMed&cmd=Retrieve&list_uids=16055682&dopt=Citation) |
| CAATBOX1 | -1021, -978, -961,  -939, -693,  -630, -603,  -535, -372,  +93, +161  [plus 10(-) strand sites] | CAAT | "CAAT promoter consensus sequence" found in *leg A* gene of pea | [2710102](http://www.ncbi.nlm.nih.gov/entrez/query.fcgi?db=PubMed&cmd=Retrieve&list_uids=2710102&dopt=Citation) |
| CACTFTPPCA1 | -1118, -1109, -830, -465, -437, -398, -154, -49, +19, +39, +54, +109 | YACT | Tetranucleotide (CACT) is a key component of Mem1 (mesophyll expression module 1) found in the cis-regulatory element in the distal region of the phosphoenolpyruvate carboxylase (*ppcA1*) | [15100398](http://www.ncbi.nlm.nih.gov/entrez/query.fcgi?db=PubMed&cmd=Retrieve&list_uids=15100398&dopt=Citation) |
| CANBNNAPA | -115 | CNAACAC | Core of "(CA)n element" in storage protein genes (*napA*); seed specificity; activator and repressor | [9002600](http://www.ncbi.nlm.nih.gov/entrez/query.fcgi?db=PubMed&cmd=Retrieve&list_uids=9002600&dopt=Citation) |
| CARGCW8GAT | -920, -773,  -719 | CWWWWWWWWG | A variant of CArG motif, with a longer A/T-rich core; Binding site for AGL15 | [12743119](http://www.ncbi.nlm.nih.gov/entrez/query.fcgi?db=PubMed&cmd=Retrieve&list_uids=12743119&dopt=Citation) |
| CCAATBOX1 | -979, -962,  -631, -604 | CCAAT | "CCAAT box" found in the promoter of heat shock protein genes; "CCAAT box" act cooperatively with HSEs to increase the hs promoter activity | [1736093](http://www.ncbi.nlm.nih.gov/entrez/query.fcgi?db=PubMed&cmd=Retrieve&list_uids=1736093&dopt=Citation); [12114568](http://www.ncbi.nlm.nih.gov/entrez/query.fcgi?db=PubMed&cmd=Retrieve&list_uids=12114568&dopt=Citation) |
| CGCGBOXAT | -452, -108 | VCGCGB | "CGCG box" recognized by *AtSR1-6* (*Arabidopsis thaliana* signal-responsive genes); Ca++/calmodulin binds to all *AtSRs*; Calmodulin-binding/CGCG box DNA-binding protein family involved in multiple signaling pathways in plants | [12218065](http://www.ncbi.nlm.nih.gov/entrez/query.fcgi?db=PubMed&cmd=Retrieve&list_uids=12218065&dopt=Citation) |
| CPBCSPOR | -851, -592 | TATTAG | The sequence critical for Cytokinin-enhanced Protein Binding in vitro | [16244912](http://www.ncbi.nlm.nih.gov/entrez/query.fcgi?db=PubMed&cmd=Retrieve&list_uids=16244912&dopt=Citation) |
| CTRMCAMV35S | 10 (-) strand sites (+225 to +243) | TCTCTCTCT | CT-rich motif (inverted GAGA) found downstream of the transcription start site of the CaMV 35S RNA; Can enhance gene expression | [15507598](http://www.ncbi.nlm.nih.gov/entrez/query.fcgi?db=PubMed&cmd=Retrieve&list_uids=15507598&dopt=Citation) |
| DOFCOREZM | -1125, -1808, -881, -387, -300, -295, +201, +269  [plus 17(-) strand sites] | AAAG | Core site required for binding of Dof proteins in maize (Z.m.); *Dof1* and *Dof2* transcription factors are associated with expression of multiple genes involved in carbon metabolism in maize | [10758479](http://www.ncbi.nlm.nih.gov/entrez/query.fcgi?db=PubMed&cmd=Retrieve&list_uids=10758479&dopt=Citation) |
| DPBFCOREDCDC3 | -12 | ACACNNG | A novel class of bZIP transcription factors, *DPBF-1* and *2* (*Dc3* promoter-binding factor-1 and 2) binding core sequence; *Dc3* expression is normally embryo-specific, and also can be induced by ABA; The *Arabidopsis* abscisic acid response gene *ABI5* encodes a bZIP transcription factor, and regulates a subset of *late embryogenesis-abundant* genes | [9225465](http://www.ncbi.nlm.nih.gov/entrez/query.fcgi?db=PubMed&cmd=Retrieve&list_uids=9225465&dopt=Citation) |
| DRE1COREZMRAB17 | -1095 | ACCGAGA | "DRE1" core found in maize (Z.M.) *rab17* gene promoter; *rab17* is expressed during late embryogenesis, and is induced by ABA | [9225468](http://www.ncbi.nlm.nih.gov/entrez/query.fcgi?db=PubMed&cmd=Retrieve&list_uids=9225468&dopt=Citation) |
| EBOXBNNAPA | -974, -961, -193, +9, +93, +276  [plus 6 (-) strand sites] | CANNTG | E-box of *napA* storage-protein gene of *Brassica napus* (B.n.), also known as RRE (R response element) | [15821875](http://www.ncbi.nlm.nih.gov/entrez/query.fcgi?db=PubMed&cmd=Retrieve&list_uids=15821875&dopt=Citation) |
| ERELEE4 | -785  [plus 2 (-) strand sites on -767 and -327] | AWTTCAAA | "ERE (ethylene responsive element)" of tomato (L.e.) E4 and carnation *GST1* genes | [8090746](http://www.ncbi.nlm.nih.gov/entrez/query.fcgi?db=PubMed&cmd=Retrieve&list_uids=8090746&dopt=Citation); [8327464](http://www.ncbi.nlm.nih.gov/entrez/query.fcgi?db=PubMed&cmd=Retrieve&list_uids=8327464&dopt=Citation) |
| GAGA8HVBKN3 | +226, +228, +230, +232, +234, +236 | GAGAGAGAGAGAGAGA | "GA octodinucleotide repeat" found in intron IV of the barley (H.v.) gene *Bkn3*; Binding site for GAGA-binding factor BBR | [12795701](http://www.ncbi.nlm.nih.gov/entrez/query.fcgi?db=PubMed&cmd=Retrieve&list_uids=12795701&dopt=Citation) |
| GAGAGMGSA1 | +226, +228, +230, +232, +234 | GAGAGAGAGAGAGAGAGA | "GAGA element" found in the promoter of the heme and chlorophyll synthesis gene *Gsa1* in soybean (G.m.) | [12177492](http://www.ncbi.nlm.nih.gov/entrez/query.fcgi?db=PubMed&cmd=Retrieve&list_uids=12177492&dopt=Citation) |
| GATABOX | -1149, -1113, -1054, -952, -911, -619, -393, -169, +131, +285, +296  [plus 4 (-) strand sites] | GATA | "GATA box" in CaMV 35S promoter; Required for high level, light regulated, and tissue specific expression; Conserved in the promoter of all LHCII type I *Cab* genes | [2535536](http://www.ncbi.nlm.nih.gov/entrez/query.fcgi?db=PubMed&cmd=Retrieve&list_uids=2535536&dopt=Citation); [12139008](http://www.ncbi.nlm.nih.gov/entrez/query.fcgi?db=PubMed&cmd=Retrieve&list_uids=12139008&dopt=Citation); [15084732](http://www.ncbi.nlm.nih.gov/entrez/query.fcgi?db=PubMed&cmd=Retrieve&list_uids=15084732&dopt=Citation) |
| GT1CONSENSUS | -952, -931,  -911, -645,  -307, -38, +184 | GRWAAW | Consensus GT-1 binding site in many light-regulated genes; GT-1 can stabilize the TFIIA-TBP-DNA (TATA box) complex; The activation mechanism of GT-1 may be achieved through direct interaction between TFIIA and GT-1; Binding of GT-1-like factors to the PR-1a promoter influences the level of SA-inducible gene expression | [8955086](http://www.ncbi.nlm.nih.gov/entrez/query.fcgi?db=PubMed&cmd=Retrieve&list_uids=8955086&dopt=Citation); [10366876](http://www.ncbi.nlm.nih.gov/entrez/query.fcgi?db=PubMed&cmd=Retrieve&list_uids=10366876&dopt=Citation) |
| GT1GMSCAM4 | -645, -307, -816  [plus 2 (-) strand sites] | GAAAAA | "GT-1 motif" found in the promoter of soybean (Glycine max) CaM isoform, *SCaM-4*; plays a role in pathogen- and salt-induced *SCaM-4* gene expression | [15310827](http://www.ncbi.nlm.nih.gov/entrez/query.fcgi?db=PubMed&cmd=Retrieve&list_uids=15310827&dopt=Citation) |
| GTGANTG10 | -843, -626,  -247, +204, +208  [plus 1 (-) strand sites] | GTGA | "GTGA motif" found in the promoter of the tobacco (N.t.) late pollen gene *g10* which shows homology to pectatelyase | [11414616](http://www.ncbi.nlm.nih.gov/entrez/query.fcgi?db=PubMed&cmd=Retrieve&list_uids=11414616&dopt=Citation) |
| HEXMOTIFTAH3H4 | -53 | ACGTCA | "hexamer motif" found in promoter of wheat (T.a.) histone genes Binding site of wheat (T.a.) nuclear protein HBP-1 (histone DNA binding protein-1); "hexamer motif" may play important roles in regulation of replication-dependent but not of replication-independent expression of the wheat histone H3 gene | [2772648](http://www.ncbi.nlm.nih.gov/entrez/query.fcgi?db=PubMed&cmd=Retrieve&list_uids=2772648&dopt=Citation); [16051676](http://www.ncbi.nlm.nih.gov/entrez/query.fcgi?db=PubMed&cmd=Retrieve&list_uids=16051676&dopt=Citation) |
| IBOX | +285 | GATAAG | Conserved sequence upstream of light-regulated genes | [2347304](http://www.ncbi.nlm.nih.gov/entrez/query.fcgi?db=PubMed&cmd=Retrieve&list_uids=2347304&dopt=Citation) |
| IBOXCORE | -952, -911,  -169, +285  [plus 3 (-) strand sites] | GATAA | Conserved sequence upstream of light-regulated genes | Terzaghi W B, Cashmore A R. Light -regulated transcription. Annual Review of Plant Physiology & Plant Molecular Biology, 1995, 46:445-474. |
| L1BOXATPDF1 | -486 | TAAATGYA | "L1 box" found in promoter of *Arabidopsis thaliana* (A.t.) *PROTODERMAL FACTOR1* (*PDF1*) gene; Involved in L1 layer-specific expression | [11439135](http://www.ncbi.nlm.nih.gov/entrez/query.fcgi?db=PubMed&cmd=Retrieve&list_uids=11439135&dopt=Citation); [15316114](http://www.ncbi.nlm.nih.gov/entrez/query.fcgi?db=PubMed&cmd=Retrieve&list_uids=15316114&dopt=Citation) |
| LEAFYATAG | -631 | CCAATGT | Target sequence of LEAFY in the intron of AGAMOUS gene in *Arabidopsis* | [12904206](http://www.ncbi.nlm.nih.gov/entrez/query.fcgi?db=PubMed&cmd=Retrieve&list_uids=12904206&dopt=Citation) |
| LTRE1HVBLT49 | -182 | CCGAAA | "LTRE-1" (low-temperature-responsive element) in barley (H.v.) *blt4.9* gene promoter | [9747801](http://www.ncbi.nlm.nih.gov/entrez/query.fcgi?db=PubMed&cmd=Retrieve&list_uids=9747801&dopt=Citation) |
| MARTBOX | +57 [plus 4 (-) strand sites] | TTWTWTTWTT | "T-Box" Motif found in SAR (scaffold attachment region; ormatrix attachment region, MAR) | [2695485](http://www.ncbi.nlm.nih.gov/entrez/query.fcgi?db=PubMed&cmd=Retrieve&list_uids=2695485&dopt=Citation) |
| MYB1AT | -235, -144, +196 | WAACCA | MYB recognition site found in the promoters of the dehydration-responsive gene rd22 and many other genes in *Arabidopsis* | [12509522](http://www.ncbi.nlm.nih.gov/entrez/query.fcgi?db=PubMed&cmd=Retrieve&list_uids=12509522&dopt=Citation) |
| MYB2CONSENSUSAT | -193, -44 [plus 1 (-) strand sites] | YAACKG | MYB recognition site found in the promoters of the dehydration-responsive gene rd22 and many other genes in *Arabidopsis* | [12509522](http://www.ncbi.nlm.nih.gov/entrez/query.fcgi?db=PubMed&cmd=Retrieve&list_uids=12509522&dopt=Citation) |
| MYBCORE | +220, +276 [plus 4 (-) strand sites] | CNGTTR | Binding site for all animal MYB and at least two plant MYB proteins *ATMYB1* and *ATMYB2*, both isolated from Arabidopsis; *ATMYB2* is involved in regulation of genes that are responsive towater stress in *Arabidopsis*; A petunia MYB protein (MYB.Ph3) isinvolved in regulation of flavonoid biosynthesis | [8312738](http://www.ncbi.nlm.nih.gov/entrez/query.fcgi?db=PubMed&cmd=Retrieve&list_uids=8312738&dopt=Citation) |
| MYBCOREATCYCB1 | -43, +188 | AACGG | "Myb core" in the 18 bp sequence which is able to activate reporter gene without leading to M-phase-specific expression, found in the promoter of *Arabidopsis thaliana* *cyclin B1:1* gene; the 18 bp sequence share homology with a sequence found in the *N.sylvestriscyclin* B1 promoter | [12139003](http://www.ncbi.nlm.nih.gov/entrez/query.fcgi?db=PubMed&cmd=Retrieve&list_uids=12139003&dopt=Citation) |
| MYBPZM | -1078 | CCWACC | 6 bp core of consensus maize P (myb homolog) binding site; Maize P gene specifies red pigmentation of kernel pericarp, cob, and other floral organs | [8313474](http://www.ncbi.nlm.nih.gov/entrez/query.fcgi?db=PubMed&cmd=Retrieve&list_uids=8313474&dopt=Citation) |
| MYBST1 | -1055, -170, +284  [plus 1 (-) strand sites] | GGATA | Core motif of MybSt1 (a potato MYB homolog) binding site; The Myb motif of the MybSt1 protein is distinct from the plant Myb DNA binding domain described so far | [7957104](http://www.ncbi.nlm.nih.gov/entrez/query.fcgi?db=PubMed&cmd=Retrieve&list_uids=7957104&dopt=Citation) |
| MYCCONSENSUSAT | -974, -961, -193, +9, +93, +276  [plus 6 (-) strand sites] | CANNTG | MYC recognition site found in the promoters of the dehydration-responsive gene *rd22* and many other genes in *Arabidopsis*; Binding site of ICE1 (inducer of CBF expression 1) that regulates the transcription of *CBF/DREB1* genes in the cold in *Arabidopsis* | [14673035](http://www.ncbi.nlm.nih.gov/entrez/query.fcgi?db=PubMed&cmd=Retrieve&list_uids=14673035&dopt=Citation); [16214899](http://www.ncbi.nlm.nih.gov/entrez/query.fcgi?db=PubMed&cmd=Retrieve&list_uids=16214899&dopt=Citation) |
| NODCON1GM | -1125 | AAAGAT | One of two putative nodulin consensus sequences | [3822835](http://www.ncbi.nlm.nih.gov/entrez/query.fcgi?db=PubMed&cmd=Retrieve&list_uids=3822835&dopt=Citation); [2338938](http://www.ncbi.nlm.nih.gov/entrez/query.fcgi?db=PubMed&cmd=Retrieve&list_uids=2338938&dopt=Citation) |
| NODCON2GM | -725, +1, +143 | CTCTT | One of two putative nodulin consensus sequences | [3822835](http://www.ncbi.nlm.nih.gov/entrez/query.fcgi?db=PubMed&cmd=Retrieve&list_uids=3822835&dopt=Citation); [2338938](http://www.ncbi.nlm.nih.gov/entrez/query.fcgi?db=PubMed&cmd=Retrieve&list_uids=2338938&dopt=Citation) |
| NTBBF1ARROLB | -464, -165, -48 | ACTTTA | *NtBBF1* (Dof protein from tobacco) binding site in Agrobacterium rhizogenes (A.r.) *rolB* gene; Required for tissue-specific expression and auxin induction | [10072394](http://www.ncbi.nlm.nih.gov/entrez/query.fcgi?db=PubMed&cmd=Retrieve&list_uids=10072394&dopt=Citation) |
| POLASIG2 | -905, -797,  -762, -602, -531 [plus 3 (-) strand sites] | AATTAAA | poly A signal found in rice alpha-amylase | [2370848](http://www.ncbi.nlm.nih.gov/entrez/query.fcgi?db=PubMed&cmd=Retrieve&list_uids=2370848&dopt=Citation) |
| POLASIG3 | -534  [plus 16 (-) strand sites] | AATAAT | Consensus sequence for plant polyadenylation signal | [3697078](http://www.ncbi.nlm.nih.gov/entrez/query.fcgi?db=PubMed&cmd=Retrieve&list_uids=3697078&dopt=Citation) |
| POLLEN1LELAT52 | -1091, -308, +177, +271 [plus 7 (-) strand sites] | AGAAA | One of two co-dependent regulatory elements responsible for pollen specific activation of tomato (L.e.) *lat52* gene | [9678581](http://www.ncbi.nlm.nih.gov/entrez/query.fcgi?db=PubMed&cmd=Retrieve&list_uids=9678581&dopt=Citation); [14976239](http://www.ncbi.nlm.nih.gov/entrez/query.fcgi?db=PubMed&cmd=Retrieve&list_uids=14976239&dopt=Citation) |
| PRECONSCRHSP70A | -491 [plus 2 (-) strand sites] | SCGAYNRNNNNNNNNNNNNNNNHD | Consensus sequence of PRE (plastid response element) in the promoters of *HSP70A* in Chlamydomonas; Involved in induction of *HSP70A* gene by both Mg Proto and light | [16971458](http://www.ncbi.nlm.nih.gov/entrez/query.fcgi?db=PubMed&cmd=Retrieve&list_uids=16971458&dopt=Citation) |
| PROXBBNNAPA | -115 | CAAACACC | "prox B (proximal portion of B-box) found in *napA* gene of Brassica napus (B.n.); CA-rich sequence, required for seed specific expression and ABA responsiveness | [10480393](http://www.ncbi.nlm.nih.gov/entrez/query.fcgi?db=PubMed&cmd=Retrieve&list_uids=10480393&dopt=Citation) |
| PYRIMIDINEBOX | -1139, -991, -103 [plus 1 (-) strand sites] | CCTTTT | Pyrimidine box found in rice (O.s.) alpha-amylase (RAmy1A) gene; Gibberellin-response cis-element of GARE and pyrimidine box are partially involved in sugar repression | [9506846;](http://www.ncbi.nlm.nih.gov/pubmed/9506846) [12226491](http://www.ncbi.nlm.nih.gov/pubmed/12226491) |
| QELEMENTZMZM13 | -1025 | AGGTCA | "Q (quantitative)-element" in maize (Z.m.) *ZM13* gene promoter; Involved in expression enhancing activity | [9747811](http://www.ncbi.nlm.nih.gov/entrez/query.fcgi?db=PubMed&cmd=Retrieve&list_uids=9747811&dopt=Citation) |
| RAV1AAT | +265 [plus 3 (-) strand sites] | CAACA | Binding consensus sequence of *Arabidopsis* (A.t.) transcription factor, *RAV1*; The expression level of *RAV1* were relatively high in rosette leaves and roots; | [9862967](http://www.ncbi.nlm.nih.gov/pubmed/9862967) |
| REALPHALGLHCB21 | -234, +197 | AACCAA | "RE alpha" found in Lemnagibba Lhcb21 gene promoter; The DNA binding activity is high in etiolated plants but much lower in green plants; Required for phytochrome regulation | [8597658](http://www.ncbi.nlm.nih.gov/entrez/query.fcgi?db=PubMed&cmd=Retrieve&list_uids=8597658&dopt=Citation) |
| ROOTMOTIFTAPOX1 | -593, -380, -136, +297 [plus 1 (-) strand sites] | ATATT | Motif found both in promoters of rolD | [7581519](http://www.ncbi.nlm.nih.gov/entrez/query.fcgi?db=PubMed&cmd=Retrieve&list_uids=7581519&dopt=Citation) |
| SEF3MOTIFGM | -965 | AACCCA | "SEF3 binding site"; Soybean (G.m.) consensus sequence found inthe 5' upstream region of beta-conglycinin (*7S globulin*) gene | [1893110](http://www.ncbi.nlm.nih.gov/entrez/query.fcgi?db=PubMed&cmd=Retrieve&list_uids=1893110&dopt=Citation) |
| SEF4MOTIFGM7S | -927, -354 [plus 1 (-) strand sites] | RTTTTTR | "SEF4 binding site"; Soybean (G.m.) consensus sequence found in 5'upstream region (-199) of beta-conglycinin (*7S globulin*) gene | [1893110](http://www.ncbi.nlm.nih.gov/entrez/query.fcgi?db=PubMed&cmd=Retrieve&list_uids=1893110&dopt=Citation) |
| SITEIIBOSPCNA | -443 | TGGTCCCAC | "Site IIb" of rice PCNA (proliferating cell nuclear antigen) gene; Binding site for two nuclear proteins, PCF1 and PCF2; Suggested to be involved in meristematic tissue-specific expression | [7599648](http://www.ncbi.nlm.nih.gov/entrez/query.fcgi?db=PubMed&cmd=Retrieve&list_uids=7599648&dopt=Citation); [9338963](http://www.ncbi.nlm.nih.gov/entrez/query.fcgi?db=PubMed&cmd=Retrieve&list_uids=9338963&dopt=Citation) |
| SP8BFIBSP8BIB | -1109 | TACTATT | One of SPBF binding site (SP8b); "SP8b" found in the 5' upstream region of three different genes coding for sporamin and beta-amylase | [7969025](http://www.ncbi.nlm.nih.gov/entrez/query.fcgi?db=PubMed&cmd=Retrieve&list_uids=7969025&dopt=Citation) |
| SURECOREATSULTR11 | +214 [plus 2 (-) strand sites] | GAGAC | Core of sulfur-responsive element (SURE) found in the promoter of *SULTR1* (a high-affinity sulfate transporter gene in *Arabidopsis*) | [15842617](http://www.ncbi.nlm.nih.gov/entrez/query.fcgi?db=PubMed&cmd=Retrieve&list_uids=15842617&dopt=Citation) |
| TATABOX5 | -945, -573, -356, -130 | TTATTT | TATA box found in the 5'upstream region of pea (*Pisumsativum*) glutamine synthetase gene | [7630938](http://www.ncbi.nlm.nih.gov/entrez/query.fcgi?db=PubMed&cmd=Retrieve&list_uids=7630938&dopt=Citation) |
| WRKY71OS | +209 [plus 4 (-) strand sites] | TGAC | "A core of TGAC-containing W-box" of, e.g., *Amy32b* promoter; Binding site of rice *WRKY71*, a transcriptional repressor of the gibberellin signaling pathway | [15047897](http://www.ncbi.nlm.nih.gov/entrez/query.fcgi?db=PubMed&cmd=Retrieve&list_uids=15047897&dopt=Citation);[10785665](http://www.ncbi.nlm.nih.gov/entrez/query.fcgi?db=PubMed&cmd=Retrieve&list_uids=10785665&dopt=Citation) |
| WUSATAg | -350 | TTAATGG | Target sequence of WUS in the intron of *AGAMOUS* gene in *Arabidopsis* | [12904206](http://www.ncbi.nlm.nih.gov/entrez/query.fcgi?db=PubMed&cmd=Retrieve&list_uids=12904206&dopt=Citation) |
| XYLAT | +267 [plus 1 (-) strand sites] | ACAAAGAA | cis-element identified among the promoters of the "core xylem gene set | [16969662](http://www.ncbi.nlm.nih.gov/entrez/query.fcgi?db=PubMed&cmd=Retrieve&list_uids=16969662&dopt=Citation) |
